# Protection against *Pseudomonas aeruginosa *lung infection in mice by recombinant OprF-pulsed dendritic cell immunization

**DOI:** 10.1186/1471-2180-10-9

**Published:** 2010-01-13

**Authors:** Lucia Peluso, Cristiana de Luca, Silvia Bozza, Antonio Leonardi, Gloria Giovannini, Alfonso Lavorgna, Gaetano De Rosa, Massimo Mascolo, Loredana Ortega De Luna, Maria Rosaria Catania, Luigina Romani, Fabio Rossano

**Affiliations:** 1 Department of Cellular and Molecular Biology and Pathology "L. Califano", University of Naples "Federico II", Via S. Pansini 5, 80131 Naples, Italy; 2 Department of Experimental Medicine and Biochemical Sciences, Section of Microbiology, University of Perugia, Via del Giochetto, 06122 Perugia, Italy; 3 Department of Biomorphological and Functional Sciences, Section of Pathology, University of Naples "Federico II", Via S. Pansini 5, 80131 Naples, Italy; 4 Oncology Referral Center of Basilicata (CROB), Regional Oncology Hospital, Rionero in Vulture, Potenza, Italy

## Abstract

**Background:**

The *Pseudomonas aeruginosa *major constitutive outer membrane porin protein F (OprF) has been shown to be a protective antigen and was previously used to activate an immunological response in a mouse model of lung pneumonia. The purpose of our study was to demonstrate the ability of mouse dendritic cells pulsed with purified or recombinant OprF to protect mice against *P. aeruginosa *infection and inflammation.

Both native (n-OprF), isolated and purified from PAO1 bacterial strain, and recombinant (histidin-conjugated) OprF (His-OprF), obtained by cloning of the *oprF *gene into the pET28a expression vector, were used to stimulate dendritic cells in vitro before adoptive transfer into prospective recipient mice with *P. aeruginosa *pulmonary infection.

**Results:**

Similar to n-OprF, His-OprF activated dendritic cells in vitro, inducing the costimulatory molecule expression as well as cytokine production. Upon adoptive transfer in vivo, porin-pulsed dendritic cells (DCs) induced Th1-mediated resistance to infection and associated inflammatory pathology caused by either the PAO1 strain or a clinically-isolated mucoid strain.

**Conclusions:**

This study highlights the pivotal contribution of DCs to vaccine-induced protection against *P. aeruginosa *infection and associated inflammation.

## Background

*Pseudomonas aeruginosa*, an ubiquitous environmental Gram-negative microrganism, is one of most important opportunistic bacteria in hospital-acquired infections [1-3]. It is responsible for acute and chronic lung infections in artificially ventilated [4] and in cystic fibrosis patients [5], and for septicemia in immunocompromised patients, including transplant and cancer patients, as well as patients with severe burn wounds. Nosocomial *P. aeruginosa *strains are characterized by an intrinsic resistance to various antimicrobial agents and common antibiotic therapies. The low permeability of the major outer membrane porins and the presence of multiple drug efflux pumps are factors that contribute to mechanisms of drug resistance in this species [6]. This high resistance leads to several therapeutic complications and is associated with treatment failure and death. The development of a vaccine against *P. aeruginosa *for active and/or passive immunization is therefore necessary as another approach to therapy.

Despite high numbers of patients who may develop *P. aeruginosa *infections and the threat of antibiotic treatment failure due to bacterial resistance, there is surprisingly no *P. aeruginosa *vaccine currently available on the market, although many attempts have been made in the past. A number of different vaccines and several monoclonal antibodies have been developed in the last decades for active and passive vaccination against *P. aeruginosa *[7]. Different antigens of *P. aeruginosa*, such as the outer membrane proteins (Oprs), LPS, toxins, pili and flagella, have been investigated as possible targets for the development of vaccines. Vaccination with outer membrane protein antigens has been shown to be efficacious against *P. aeruginosa *infection in a number of studies using killed whole cells [8], purified outer membrane preparations [9], isolated outer membrane proteins [10-12], protein fusions, or synthetic peptides representing protective epitopes [13]. The *P. aeruginosa *major constitutive porin protein, OprF, which has previously been shown to be antigenic [10,14] and has high homology among *Pseudomonas *strains [11,15], was also chosen as a vaccine target [16]. This protein has been shown to provide protection in a mouse model of systemic infection [10], a mouse burn infection model, and rodent models of acute [17] and chronic lung infection [11].

While many of experimental vaccines and monoclonal antibodies have been tested in preclinical trials, few have reached clinical phases because it is difficult to study cystic fibrosis patients, in which improved antibiotic therapy impaired a proper evaluation of the vaccine's efficacies [7] and none of these vaccines has obtained market authorization [8]. New promising perspectives for the development of vaccination strategies against various types of pathogens are the use of antigen-pulsed dendritic cells (DCs) as biological immunizing agents [18-20].

DCs are specialized antigen-presenting cells that play a dual role in inducing adaptive immune responses to foreign antigens and in maintaining T cell tolerance to self [21]. Although there are still numerous controversial and unresolved issues surrounding DC-mediated immune responses against pathogens [22], the role of DCs in immunity to *P. aeruginosa *is undisputed [23]. Moreover, DCs have a central role in developing new vaccine strategies due to some prominent features, such as location, antigen handling, maturation, and subsets [21,24].

We designed and tested the efficacy of OprF-pulsed DCs for a vaccine based upon adoptive transfer in mice with *P. aeruginosa *infection. To overcome the problem of quantity and purity related to the purification of OprF from bacterial outer membrane, we resorted to recombinant OprF, C-terminal part of which carries an important protective epitope [25]. The results reported in this paper demonstrate the ability of mouse DCs pulsed with purified or recombinant OprF to protect mice against *P. aeruginosa *infection and inflammation.

## Results and Discussion

### Native or recombinant OprF activate DCs in vitro

**Figure 1:**
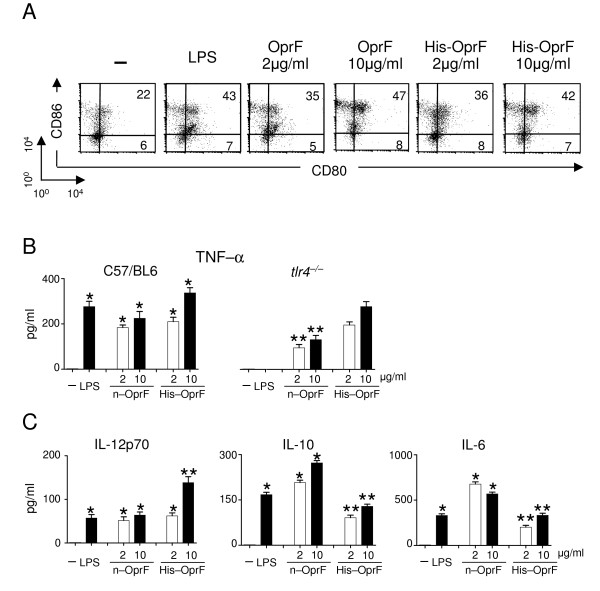
**Activation of murine dendritic cells by OprF**. Purified splenic dendritic cells (DCs) were pulsed with LPS (10 μg/ml), native (n) or recombinant (His) OprF at different concentrations for 18 hrs before the assessment of costimulatory molecule expression (A) and cytokine production (B and C). FACS analysis was done by staining with FITC and PE-conjugated mAbs to costimulatory molecules. Number represent percent of positive cells. Cytokine levels were determined in the culture supernatants by cytokine-specific ELISA. * Indicates *P *< .05 (cytokine production by LPS- or porin-pulsed *versus *unpulsed (-) DCs). ** Indicate *P *< .05 (cytokine production by n-OprF-pulsed *tlr4*^-/- ^DCs *versus *n-OprF-pulsed WT DCs only and His-OprF-pulsed DCs *versus *n-OprF-pulsed DCs).

To assess the immunogenic capacity of native or recombinant OprF, we evaluated levels of costimulatory antigen expression (CD80 and CD86) and cytokine production of DCs pulsed with different concentrations (2 and 10 μg) of either native or recombinant OprF or LPS, as a positive control. Similar to LPS, both porins increased CD86 and CD80 expression in a dose-dependent manner (Fig. 1A). Class II MHC antigen expression was also significantly increased by 10 μg/ml of both porins (from 19 to 47, 43 and 45% of positive cell in unpulsed DCs *versus *LPS-, n-OprF- or His-OprF-pulsed DCs). In terms of cytokine production, both porins induced equivalent levels of TNF-α production, which was partially Toll like receptor (TLR)4-dependent for the native porin but almost totally TLR4-independent for the recombinant porin (Fig. 1B), a finding confirming the absence of LPS contamination in the His-OprF preparation. Interestingly, levels of IL-12p70 production were higher and those of IL-10 and IL-6 lower in DCs stimulated with the recombinant porin as compared to the native porin (Fig. 1C), a finding suggesting the superior capacity of the recombinant OprF to activate DCs for Th1 priming.

### OprF-pulsed DCs protect mice from PAO1 infection

**Figure 2:**
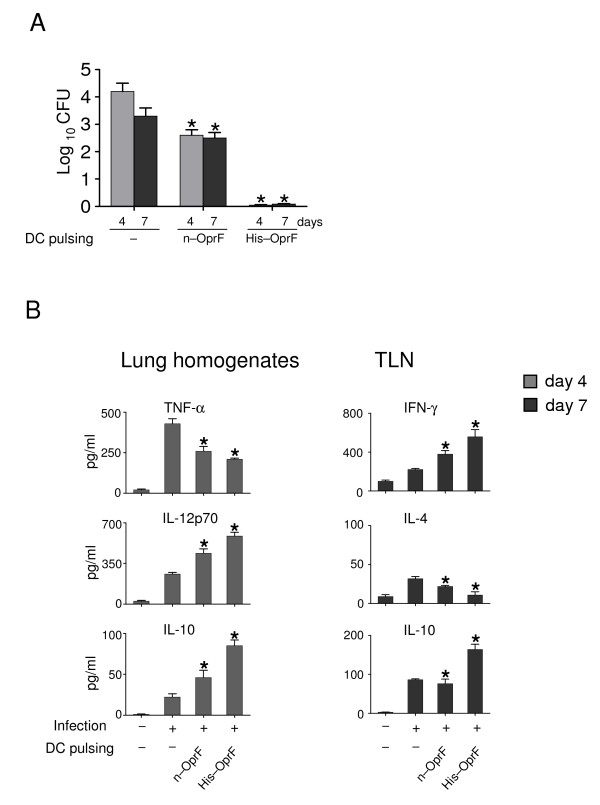
**OprF-pulsed DCs protect mice from infection with the PAO1 strain**. Splenic 10^5 ^dendritic cells (DCs), either unpulsed (-) or pulsed as in legend to figure 1, were administered into recipient mice intraperitoneally a week before the intranasal injection of 3 × 10^7 ^*P. aeruginosa *PAO1 strain. (A) Resistance to infection was assessed in terms of CFU at different days after the infection and (B) cytokine production in lung homogenates and culture supernatants of total cells from TLNs stimulated with plate bound anti-CD3e (2 μg/ml) and anti-CD28 (2 μg/ml) for 72 hours. Results are expressed as mean ± SE. * Indicates *P *< .05, mice receiving pulsed *versus *unpulsed (-) DCs. In C, - and + alone indicate uninfected and infected mice, respectively.

Based on these results, we assessed the capacity of DCs pulsed with either porin to immunize mice against *P. aeruginosa *lung infection. To this purpose, porin-pulsed DCs were administered to mice a week before the intranasal infection with the PAO1 strain. Mice were monitored for bacterial growth, lung inflammatory pathology and cytokine production locally in the lung (at 4 days after the infection) or in the thoracic lymph nodes (TLNs, at 7 days after infection). The results (Fig. 2A) showed that the adoptive transfer of DCs pulsed with n-OprF exerted significant protection in terms of reduced bacterial growth, both at 4 and 7 days after the infection. No effects on bacterial clearance was observed upon adoptive transfer of unpulsed DCs.	Interestingly, an even higher bacterial clearance was observed upon adoptive transfer of DCs pulsed with His-OprF, being the bacterial growth dramatically reduced as early as 4 days after the infection.

As a similar degree of protection in terms of reduced bacterial clearance was also observed upon infecting the mice intratracheally (data not shown), we concluded that the lower airways of the lung are the sites of both the induction and expression phase of the DC-induced resistance against *P. aeruginosa*.

To correlate vaccine-induced resistance with pattern of inflammatory and Th cytokine production in mice with infection, levels of pro-inflammatory (TNF-α/IL-12p70) or anti-inflammatory (IL-10) cytokines were measured in the lung homogenates and those of Th1 (IFN-γ) or Th2 (IL-4/IL-10) in antigen-stimulated TLNs. The results show that levels of TNF-α were significantly reduced whereas those of IL-12p70 and IL-10 both increased in vaccinated mice (Fig. 2B). In the TLNs, the levels of Th1/IFN-γ production were increased in mice vaccinated with DCs pulsed with either porin, while those of Th2/IL-4 were decreased, particularly with the His-OprF-pulsed DCs. Interestingly, mice vaccinated with His-OprF-pulsed DCs also showed increased levels of IL-10 production. As in cystic fibrosis (CF) patients priming of the cellular immune system towards a Th1-like pattern seems to be of potential advantage [26], while pulmonary Th2 responses are seen in CF patients with *Pseudomonas *pneumonia [27], our data suggest that vaccine-induced resistance correlates with the activation of protective Th1 cell responses and decrease of non-protective Th2 responses.

**Figure 3:**
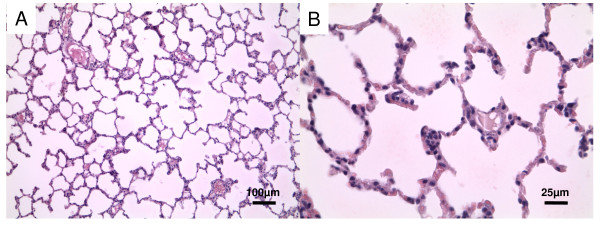
**Lung sections from uninfected mice**. Lung sections were hematoxylin-eosin stained. A - magnification ×10. B - magnification ×40.

**Figure 4:**
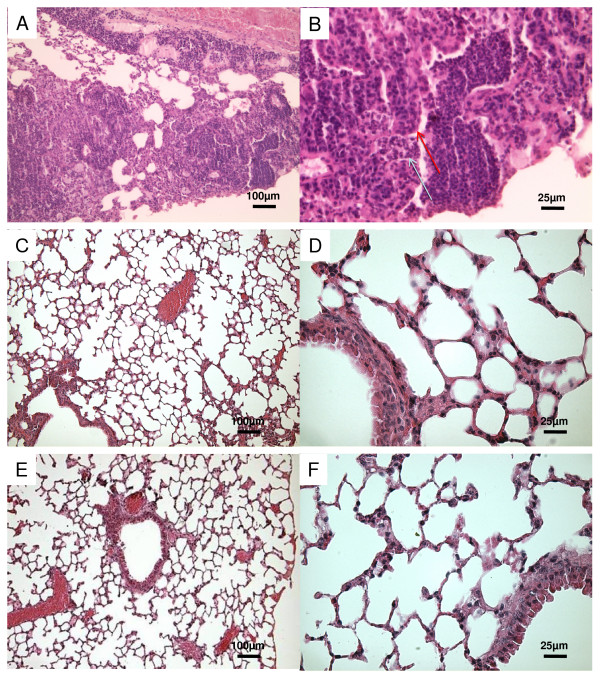
**Lung sections of mice vaccinated with OprF-pulsed DCs and infected with PAO1 strain**. Histopathology at 7 days after infection. Lung sections A-B from infected mice show the involvement of bronchioles and of the alveolar space by an inflammatory infiltrate predominantly consisting of neutrophils filling most of bronchioles (red arrow: bronchial epithelium; blue arrow: neutrophilic infiltrate); the lungs sections from mice vaccinated with n-OprF-pulsed DCs (C-D) and His-OprF-pulsed DCs (E-F) show a great reduction of inflammatory cell recruitment. Lung sections were hematoxylin-eosin stained. A-C-E magnification ×10. B-D-F magnification ×40.

To correlate these findings with levels of pulmonary inflammation, we evaluated sections of lungs from uninfected, infected or vaccinated mice for inflammatory cell recruitment and lung injury (Fig 3 and 4). In the Fig. 4A and 4B haematoxylin-eosin sections from mice infected with PAO1 strain show the presence of lung parenchyma, with an evident inflammatory infiltrate, mainly constituted of polymorphous granulocytes, involving small bronchi, bronchioles, and alveoli, up to the formation of abscesses with tissue necrosis.

In contrast, inflammatory cell recruitment was greatly reduced in the lungs of mice vaccinated with n-OrpF-pulsed DCs (Fig 4C and 4D) or His-OprF-pulsed DCs (Fig. 4E and 4F).

### OprF-pulsed DCs protect mice from infection with the clinical isolate

**Figure 5:**
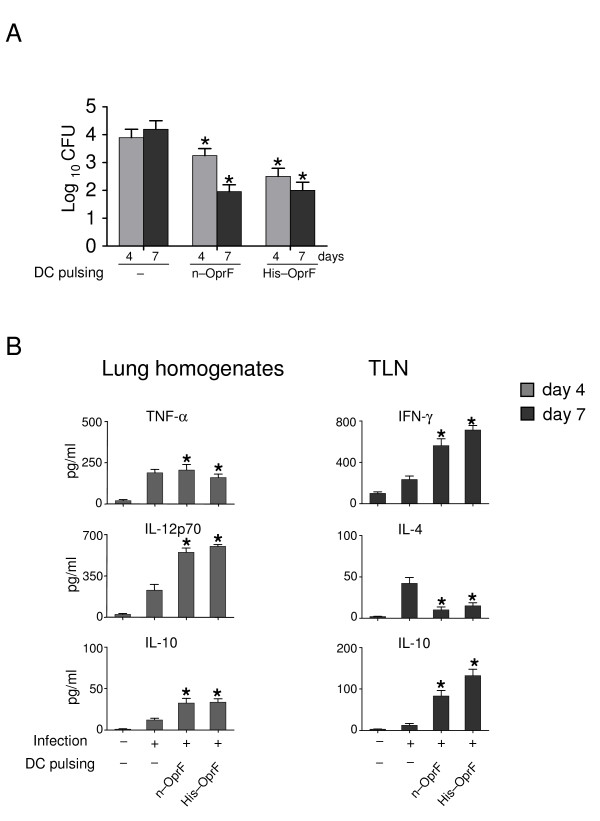
**OprF-pulsed DCs protect mice from infection with the clinical isolate**. Splenic DCs were pulsed and administered as in legend to figure 1. Mice were infected intranasally with 3 × 10^7 ^*P. aeruginosa *mucoid strain. (A) Resistance to infection and (B) cytokine production in lung homogenates and culture supernatants of TLNs were assessed as in legend to Figure 2. * Indicates P < .05 (mice receiving pulsed *versus *unpulsed (-) DCs). In C - and + alone indicate uninfected and infected mice, respectively.

**Figure 6:**
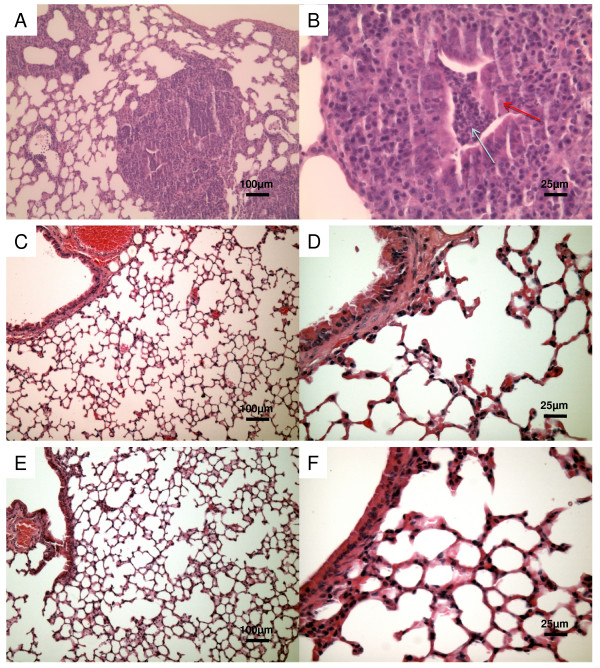
**Lung sections of mice vaccinated with OprF-pulsed DCs and infected with clinical isolate**. Lung sections A-B representing histologic pictures of pneumonia similar to those described in fig. 4 are shown (red arrow: bronchial epithelium; blue arrow: neutrophilic infiltrate). Lung sections from mice vaccinated with n-OprF-pulsed DCs (C-D) and His-OprF-pulsed DCs (E-F) show a lung in which inflammatory cell recruitment was greatly reduced. Lung sections were hematoxylin-eosin stained. A-C-E magnification ×10. B-D-F magnification ×40.

Because chronic lung infections with *P. aeruginosa *are associated with the diversification of the persisting clone into different morphotypes [28] and *P. aeruginosa *isolates from chronic CF lung infections are phenotypically quite distinct from those causing acute infections in other settings [29], we assessed whether the vaccinating potential of porin-pulsed DCs would extend to a mucoid strain isolated from CF patients. To this purpose, mice were treated, infected and evaluated for microbiological and immunological parameters as above. Figures 5A, B and 6 show the cumulative results of these experiments. Consistent with the high virulence of mucoid bacterial strains [30], the clearance of the bacteria from the lung was delayed, as judged by the high level of bacterial colonization at 7 days after infection (Fig. 5A). Nevertheless, treatment with either type of pulsed DCs significantly reduced bacterial growth, although to a lesser extent compared to PAO1-infected mice (Fig. 5A). Although levels of Th1 cytokines (IL-12p70/IFN-γ) were significantly higher and those of Th2/IL-4 lower in DCs-vaccinated mice as compared to untreated mice, levels of TNF-α were not significantly decreased in DCs-treated *versus *untreated mice. Moreover, although increased if compared to untreated mice, levels of IL-10 were not as high as those induced in PAO1-infected mice (Fig. 5B). Lung inflammatory cell recruitment was significantly reduced by treatment with either type of pulsed DCs, although to a lesser extent compared to PAO1-infected mice (Fig. 6). Together, our data indicate that porin-pulsed DCs may induce immune protection against pulmonary infection by *P. aeruginosa *with a significant reduction of inflammation.

It is believed that the initial site of colonization by *P. aeruginosa *is localized to the upper respiratory epithelium; therefore, inducing mucosal immunity to this pathogen appears to be an ideal strategy for the prevention of infection. Previous studies suggested that systemic immunity, from either oral vaccination [8] or i.p. vaccination [31] with *P. aeruginosa *vaccine constructs, was as effective as mucosal delivery of the vaccine in a mucosal challenge.

We found here that peripheral delivery of porin-pulsed DCs also resulted in active immunization against *Pseudomonas *pneumonia. Protection occurred against pneumonia induced by either intranasal or intratracheal delivery of the bacteria, a finding consistent with the above-mentioned studies and confirming that peripheral immunization may result in mucosal and parenchymal protection at distal sites.

Protection was associated with increased bacterial clearance, decreased inflammatory pathology and the occurrence of Th1 immunity in the draining lymph nodes. Although antibodies have a crucial role in protection against *P. aeruginosa *infection, cell-mediated immunity is also important in the clearance of the bacterium. The observation that the occurrence of a protective Th1 reactivity coexisted with the detection of significant levels of IL-10 is intriguing. It is known that high levels of IL-10 are associated with protection in patients with CF and IL-10 is required for the induction of regulatory T cells dampening inflammation in infections [32]. Whether IL-10 produced in DCs-vaccinated mice may serve to support the growth of regulatory T cells preventing prolonged inflammation is an attractive working hypothesis.

## Conclusions

There is surprisingly no *P. aeruginosa *vaccine currently available on the market, although many attempts have been made in the past. This raises the question as to whether *P. aeruginosa *is an antigenically variable microorganism that can escape immune recognition and/or induce immunological non-responsiveness as is seen with other bacteria such as *Borrelia*, *Bordetella *or *Neisseria*. Because the organism has the ability to undergo phenotypic variation due to changing environmental conditions such as in the airways of CF patients [29], the highly conserved antigens such as Oprs represent ideal candidates for vaccines. However, despite highly efficient technologies to express proteins and to purify protein and carbohydrate antigens in high yields under good manufacturing practices standards, the lack of a protective *P. aeruginosa *vaccine is a reality. Our study would suggest that the use of porin-pulsed DCs may represent a possible candidate vaccine against *Pseudomonas *infection. As DCs conferred protection against both the conventional PAO1 strain and the more virulent mucoid strain, this finding highlights the potential of DCs to overcome the mucin-dependent negative regulation of immune responses to *P. aeruginosa *[33].

Confirming the efficacy of several tested Opr vaccine preparations in generating protection against different *P. aeruginosa *challenges in preclinical studies [9], OprF-pulsed DCs not only induced Th1 resistance to the infection but also ameliorate inflammatory pathology. This finding is of relevance considering the contribution of a self-sustaining cycle of airway obstruction, infection, and inflammation to lung disease in CF [34]. CF lung disease is characterized by neutrophilic airway inflammation, increased expression of proinflammatory cytokines, and infection by a narrow repertoire of bacterial pathogens, with *P. aeruginosa *and *Burkholderia cepacia *complex being the most clinically significant pathogens. Current therapy for CF lung disease relies on antibiotics to treat bacterial infection combined with airway clearance strategies to mobilize viscid secretions. However, anti-inflammatory therapy has been shown to be beneficial for patients with CF [34], especially for younger patients with mild disease. Recent data indicate that TLR4- and flagellin-induced signals mediate most of the acute inflammatory response to *Pseudomonas *[35]. The fact that DCs activation by recombinant OprF occurred independently of TLR4 would suggest that avoiding the damaging inflammatory pathway to the bacterium may be of benefit in vaccine-induced protection. Overall, our study points to the successful combination of recombinant porins and DCs for vaccine-induced protection in the relative absence of innate danger signals. However, much needs to be done to work out principles that govern the regulation of the human immune system in vivo in patients with pneumonia, including the immunobiology of DCs in immune resistance to *Pseudomonas*.

## Methods

### Bacterial strains and growth conditions

The strain of *P. aeruginosa *PAO1 was purchased from the American Type Culture Collection, Rockville, MD. (ATCC, BAA-47). A clinical strain, isolated from a CF patient, was obtained from the Diagnostic Unit of Microbiology of the University of Naples "Federico II". The bacteria were grown on 2% proteose peptone (PP2) and 0.5% NaCl. Overnight cultures grown under continuous shaking at 37°C, were diluted 10- to 20- fold into fresh medium at 37°C to an optical density of 0.6-0.8 (600 nm).

### Mice

Female C57BL/6 mice, 8-10 wk old, were purchased from Charles River (Calco, Italy). Homozygous *Tlr4^-/- ^*mice on a C57BL/6 background were bred under specific pathogen-free conditions at the Animal Facility of Perugia University, Perugia, Italy [36]. Experiments were performed according to the Italian Approved Animal Welfare Assurance A-3143-01.

### Purification of native porin F (OprF) from *P. aeruginosa*

The porin was isolated and purified from PAO1 bacterial strain following the method described by Hancock R.E.W (Hancock Laboratory Methods, Department of Microbiology and Immunology, University of British Columbia, British Columbia, Canada, http://www.cmdr.ubc.ca/bobh/methods/PORINPURIFICATION.html). Briefly, bacteria were grown overnight at 37°C; fresh inoculum was added the day after and grown until logarithmic phase. Bacteria were harvested and resuspended in 20% sucrose, 10 mM Tris-HCl, pH8, in the presence of DNaseI (50 μg/ml). The cells were broken by a French Press at 15,000 psi three times and then sonicated (power 35/5 cycles/30 seconds). The lysate was applied to the top of a 2-step sucrose gradient (72% and 52%) and centrifuged at 58,357 g overnight at 4°C. The day after, the outer membranes were collected and washed by centrifugation at 142,743 g/1 h/4°C. The proteins of the outer membrane were purified by solubilization with 2% Triton X-100, 20 mM TrisHCl, pH8, and then with 2% Triton X-100, 20 mM TrisHCl, pH8, 10 mM EDTA, to remove all remaining bound LPS and phospholipids. At each passage, the pellet was sonicated at a probe intensity of 35/30 sec and then centrifuged at 145,424 g/1 hr/4°C. The fractions, solubilized with 2% Triton X-100, 20 mM TrisHCl, pH8, were centrifuged 145,424 g/1 hr/4°C and the supernatant was loaded on a DEAE-Sephacel column, equilibrated with 0.2% Triton X-100, 20 mM TrisHCl, pH8, 10 mM EDTA (column buffer). OprF was eluted using a 0.1 M - 0.3 M NaCl linear gradient. The porin preparation was run on a gel-filtration column (Amersham Biosciences), (column buffer was: 0.25% SDS, 10 mM NaCl, 5 mM EDTA, 0.05% β-mercaptoethanol). The purity of OprF was checked by SDS-PAGE followed by Western blotting with the MA7-7 at high specificity monoclonal antibody [37] (kindly gifted by Dr R.E.W Hancock). *Limulus *amoebocyte lysate (LAL) assay [38] was performed to evaluate LPS contamination (100 pg/μg porins) in native porin preparation.

### Preparation of recombinant OprF (His-OprF)

Genomic DNA was extracted from *P. aeruginosa *PAO1 strain and the *oprF *sequence was amplified by PCR with specific primers: 5'-CGCGGATCCAAACTGAAGAACACCTTAGGCGTTGTC-3' (Fw) and 5'-CCCAAGCTTTTACTTGGCTTCGGCTTCTACTTCGGC-3' (Rev). The *oprF *gene fragment was cloned (BamHI and Hind III) into the pET28a expression vector (Novagen), that has an His_6 _affinity tag at the 5' end of the polylinker that functions as a high affinity nickel-binding domain in the translated protein. To be sure that all the OprF nucleotide sequence was completely cloned, the plasmid was sequenced by automated sequencing using Sanger's method and the sequence was compared with the sequence reported in GenBank. The Qiagen expression host cells, *E. coli *BL21, were made competent and transformed with the resulting plasmid pET28a-*oprF*. Expression of recombinant OprF (His-OprF) was induced by the addition of isopropyl-β-D-thiogalactoside (IPTG) (Sigma; 1 mM final concentration). *E. coli *BL21 cells were harvested by centrifugation and His-OprF was purified by denaturing conditions on a nickel-nitrilotriacetic acid affinity chromatography gel matrix (Sigma Aldrich). The recombinant protein purification was performed by denaturing conditions in four steps, as follows: solubilization with 8 M urea, 0.1 M NaH_2_PO_4_, 0.01 M TrisHCl, pH8; washing with 8 M urea, 0.1 M NaH_2_PO_4_, 0.01 M TrisHCl, pH 6.3 and 8 M urea, 0.1 M NaH_2_PO_4_, 0.01 M TrisHCl, pH 5.9; eluation of the interested protein with 8 M urea, 0.1 M NaH_2_PO_4_, 0.01 M TrisHCl, pH 4.5. Pure His-OprF was solubilized in 0.25% SDS, 5 mM EDTA. LPS contamination was revealed on SDS_PAGE gels stained with silver nitrate [39] and quantified by *Limulus *amoebocyte lysate (LAL) assay [38]. Recombinant OprF preparation was completely free from LPS contamination. Moreover, the purity of OprF was checked by SDS-PAGE, followed by Western blotting using MA7-7 [37] an high specific monoclonal antibody (kindly gifted by Dr R.E.W Hancock).

### Mice infection with *P. aeruginosa*

C57/BL6 mice were intranasally infected with the non lethal dose of 3 × 10^7 ^colony forming units (CFU) of *P. aeruginosa *PAO1 strain or the clinically isolated strain, as from preliminary experiments. At day 4 and day 7 of infection, mice were sacrificed and lung tissues were homogenized in PBS buffer containing soybean trypsin inhibitor. For the bacterial counts, 50 μl dilutions of the homogenate were plated on trypticase soy agar plates and then incubated for 24 hrs at 37°C. CFU, quantified by serial plating on trypticase soy agar plates, were determined in the lung at 4 or 7 days after infection. The results (means ± standard errors) are expressed as CFU/organ. The remaining homogenate was centrifuged at 16,060 g/30 min/4°C and the supernatant was stored at -80°C for cytokine determination.

### Histology

Lungs were excised en bloc and inflation fixed in 4% paraformaldehyde in PBS. The lungs were then embedded in paraffin, and sections were cut and stained with hematoxylin and eosin using standard techniques.

### Isolation of DCs

DCs were purified from spleens by magnetic-activated sorting using CD11c MicroBeads and MidiMacs (Miltenyi Biotec), in the presence of EDTA to disrupt DCs-T cell complexes [36]. Cells were >99% CD11c^+^, < 0.1% CD3^+^, and appeared to consist of 90-95% CD8^-^, 5-10% CD8^+^, and 1-5% B220^+ ^cells.

### Antigen pulsing of DCs and mice immunization

DCs were pulsed for 2 hrs at 37°C with native OprF or with recombinant His-OprF (10 μg/1 × 10^6 ^cells). Pulsed DCs (5 × 10^5^) were extensively washed before being administered intraperitoneally a week before the intranasal infection with either strain of *P. aeruginosa*. Aliquots of DCs were assessed for cytokine production and costimulatory antigen expression after 18 hrs of culture. Positive controls included DCs stimulated with 10 μg/ml ultra-pure lipopolysaccharide (LPS) from *Salmonella minnesota *Re 595 (Labogen S.r.l., Rho, Milan, Italy).

### Cytokine assays

The cytokine levels in culture supernatants of pulsed-DCs, in lung homogenates (at 4 days after infection) or culture supernatants from thoracic lymph nodes (TLNs, at 7 days after infection) were measured by ELISA (R&D Systems, Inc., Space Import-Export srl, Milan, Italy). The detection limits (pg/ml) of the assays were <10 for IFN-γ, <32 for TNF-α <3 for IL-10, <16 for IL-12p70 and <7 for IL-6.

### Flow cytometry

Staining was done as described [36]. For double staining, DCs were sequentially reacted with saturating amounts of FITC-conjugated anti-CD80 and PE-conjugated anti-CD86 mAb from BD Pharmingen (CD80 and CD86). Cells were analyzed with a FACScan flow cytofluorimeter (Becton Dickinson) equipped with CELLQuest™ software. Control staining of cells with irrelevant Ab was used to obtain background fluorescence values. Data are expressed as a percentage of positive cells over total cells analyzed. Flow cytometry was used to determine the purity of isolated cells.

### Statistical analysis

Data were analyzed on PC using InStat version 2.01 and GraphPad Prism version 4.0 statistical packages (GraphPad Software). The double-tailed Student's *t *test was used to compare the significance of differences between groups. A value of P < 0.05 was considered significant. The data reported are either from one representative experiment out of three independent experiments (FACS analysis) or pooled from three to five experiments, otherwise. The in vivo groups consisted of 6-8 mice/group.

## Abbreviations

CF: Cystic Fibrosis; CFU: colony forming units; DCs: dendritic cells; His-OprF: histidin-conjugated outer membrane protein F; TLNs: thoracic lymph nodes; OprF: Outer membrane protein F.

## Competing interests

The authors declare that they have no competing interests.

## Authors' contributions

LP has given an important contribution to the elaboration of paper. CdL, SB, AL, LODL and MRC gave important contributions in the order to design of the paper and to draft of manuscript. GG and AlL have cooperated for technical assistance. GDR and MM have studied histopathology features. FR and LR conceived the study participating to its scientific design. All authors read and approved the final manuscript.
